# Supplementary material for: Pilot Study on Risk Perception in Practices with Medical Cyclotrons in Radiopharmaceutical Centers in Latin American Countries: Diagnosis and Corrective Measures
Source: Int J Environ Res Public Health. 2025 Dec 18;22(12):1885. doi: 10.3390/ijerph22121885 (PMC12732775; doi:10.3390/ijerph22121885)
Supplement: Supplementary file 1 [file ijerph-22-01885-s001.zip › ijerph-3909116-supplementary.pdf]

QUESTIONNAIRE TO ASSESS RISK PERCEPTION IN WORKERS OF CENTERS FOR THE PRODUCTION AND CLINICAL USE OF RADIOPHARMACEUTICALS

Familiarity

1. Years of work with ionizing radiation

1. 1 to 5
2. 5 to 10
3. more than 10

Understanding Risk

2. Know the dangers that are exposed to work

1. No, I am not aware of the dangers to which I may be exposed.
2. I am aware of some dangers, but I would like to know more about them.
3. Yes, I am aware of the dangers that exist.

3. Know the measures to deal with a radiological incident or emergency (target rupture, loss of bunker pressure, vial rupture, radiopharmaceutical spillage, etc.)

1. No, I am not familiar with the measures to deal with a radiological incident or emergency.
2. I have heard of some measures, but I do not have a complete knowledge about how to deal with a radiological incident or emergency.
3. Yes, I know the measures and procedures in place in the event of radiological incidents or emergencies.

4. Do you feel that you have received sufficient training regarding the dangers of your work?

1. No, I don't think I have received enough training.
2. I'm not sure, I'd like to receive more information about the dangers of my job.
3. Yes, I consider that I have received sufficient training regarding the dangers of my work.

5. Have you received training where radiological emergency drills are conducted?

1. No, I have never received training in radiological emergency drills.
2. Yes, I have been trained in radiological emergency drills once.
3. Yes, I have been trained in radiological emergency drills several times.

6. *Know the means available to solve an incident (stopping irradiation, cyclotron, rupture of a target, loss of pressure in the synthesis cells, spillage of liquids, etc.)*

1. No, I am not aware of the means available to solve an incident.
2. Some, I am aware of some means available to solve an incident.
3. Yes, I know all the means available to solve an incident.

Uncertainty

7. *Do you think that all the events that could cause an incident or accident have been sufficiently studied?*

1. No, there is still a lot to study in this area.
2. I think so, but there is always room for further research.
3. Yes, all possible events have been studied.

Voluntarism

8. What degree of willingness to solve an incident or accident (leakage of radioactive material in gaseous form, spillage of a vial with radioactive material, etc.)

1. Casualty
2. Regular
3. Loud

9. You find it difficult or uncomfortable to use the means to solve an incident

1. Yes, sometimes the means can be complicated or uncomfortable to solve an incident.
2. It depends on the type of incident, sometimes yes and sometimes not.
3. No, I always use the means to solve any type of incident and they are very useful to me.

Personal involvement

10. What degree of impact on your health do you expect as a result of your work activity?

1. Little affectation,
2. Acceptable affectation,
3. High impact

11. *You are aware of the level of possible impact on your health when faced with an incident or accident*

1. It doesn't affect me,
2. acceptable affectation,
3. high impact

Controllability

12. *Do you consider that all the risks to which you are subjected in your profession are under control?*

1. no control,
2. medium control,
3. Full Control

Employment

13. *Do you work only in this center or do you work in other production centers?*

1. In this center
2. In other centers, in addition to this

Catastrophic potential

14. *What level of severity do you attribute to interventions in incidents in which you are exposed to irradiation?*

1. Casualty
2. stocking
3. loud

15. *Do you consider that the severity of operational incidents involving radiation-related equipment is like other types of incidents?*

1. are less severe
2. are similar,
3. are more serious

Past history of accidents

16. *Knows about accidents from their own experience or through studies*

1. Studies
2. Mixed

3. Great experience of our own

17. *Has participated in interventions in accidental situations*

1. never
2. ever
3. several times

Immediacy of consequences

18. *Considers that the consequences of irradiation are more important in the immediate or long-term*

1. Late
2. mixed
3. immediate

Reversibility of consequences

19. *Considers that the psychological consequences of an accident are reversible*

1. Non-recoverable,
2. Moderately recoverable,
3. Fully recoverable

20. *Considers damage to be reversible in the event of radioactive exposure*

1. Irreversible
2. reversible with sequelae,
3. Fully reversible

Panic

21. *Are you afraid of intervention in a radioactive emergency?*

1. No, I am not afraid of it.
2. Something, I feel a little afraid.
3. Yes, it scares me a lot.

Effects on generations

22. *Can the consequences of an accident influence future generations?*

1. Never

2. possibly
3. surely

23. *Can the consequences of any radiological accident relate to the production and/or use of PET radiopharmaceuticals influence the environment?*

1. I don't think so.
2. I think it depends on multiple factors.
3. Yes, definitely.

24. *In the event of an incident in the cyclotron or synthesis modules, could genetic effects be expected in the exposed population?*

1. never
2. possibly
3. surely

Identity of the victims

25. *Do you know of any victims of a radioactive accident in the production or use of radiopharmaceuticals or is your knowledge reduced statistics?*

1. know only statistics
2. Know statistics and victims
3. Knowledge of victims prevails over statistics

Proceeds

26. *How do you consider the personal or collective benefits obtained from your work?*

1. Low
2. Suitable
3. very high

Trust in institutions

27. *Do you consider the institutions that manage radiation protection to be credible? (dosimetry, audits, inspections)*

1. Total disbelief
2. Medium credibility
3. High credibility

Supervisors' response

28. *Supervisors require compliance with all radiation protection measures*

1. little
2. sometimes,
3. always

Response from colleagues

29. *Your colleagues take all the protective measures in the execution of the practice*

1. almost never
2. sometimes
3. always

Demand

30. *How do you consider the work rhythms, shifts and working conditions of your work?*

1. low workload,
2. acceptable workload,
3. Very high workload
